# Ethanol extract from *Argyreia acuta* Lour. leaves exhibit analgesic, antipyretic, and anti-inflammatory effects in mouse models

**DOI:** 10.5114/bta/204527

**Published:** 2025-06-30

**Authors:** Tran Thi Phuong Nhung, Le Pham Tan Quoc, Dang Thi Kim Thy

**Affiliations:** Institute of Biotechnology and Food Technology, Industrial University of Ho Chi Minh City, Ho Chi Minh City, Vietnam

**Keywords:** analgesic effects, antipyretic effects, anti-inflammatory effects, ethanol extract, traditional medicinal plants

## Abstract

**Background:**

*Argyreia acuta* has traditionally been used for its analgesic, antipyretic, and anti-inflammatory properties; however, scientific validation of these effects remains limited. This study aimed to evaluate the pharmacological potential of ethanol extract from *A. acuta* leaves (AAEE) in murine models of pain, fever, and inflammation.

**Materials and methods:**

The pharmacological properties of *A. acuta* leaf extract were assessed. Analgesic activity was evaluated using a hot plate and tail-flick assays, while antipyretic effects were tested via a yeast-induced pyrexia model. The anti-inflammatory potential was investigated through carrageenan-induced paw edema and by quantifying pro-inflammatory mediators, including TNF-α, IL-1β, IL-6, COX-2, and PGE_2_. Histopathological analysis of paw tissues was performed to confirm inflammatory changes.

**Results:**

AAEE exhibited significant, dose-dependent analgesic effects, as indicated by prolonged latency times and increased pain inhibition (*p* < 0.05), with the 200 mg/kg dose showing the greatest efficacy. In the antipyretic model, AAEE at 200 mg/kg reduced rectal temperature to 36.93°C, corresponding to an inhibition rate of 82.61% (*p* < 0.05). The extract significantly reduced paw edema (41.39% inhibition at 200 mg/kg) and markedly lowered levels of TNF-α, IL-1β, IL-6, COX-2, and PGE_2_ (*p* < 0.05). The histological analysis supported these findings, revealing decreased edema and inflammatory cell infiltration in treated groups.

**Conclusions:**

These findings provide scientific support for the traditional use of *A. acuta*, demonstrating its significant analgesic, antipyretic, and anti-inflammatory activities. AAEE may represent a promising natural therapeutic agent for treating pain, fever, and inflammation.

## Introduction

*Argyreia acuta* Lour., a member of the Convolvulaceae family, is widely distributed in tropical and subtropical regions, including Southeast Asia, India, and China (Zhu et al. 2001). Traditionally, this plant has been used in folk medicine for its purported analgesic, antipyretic, and anti-inflammatory properties (Li et al. 2021). Pain, fever, and inflammation are fundamental physiological responses to tissue injury or infection and are closely associated with various acute and chronic pathological conditions. These symptoms not only diminish the quality of life but also place a significant burden on healthcare systems (Qi et al. 2024). Chronic pain, in particular, can impair mobility and productivity, while unresolved inflammation and prolonged fever are linked to serious conditions such as arthritis, cardiovascular diseases, and immune dysfunction (Cohen et al. 2022).

Several species within the Convolvulaceae family, including *Ipomoea pes-caprae* and *Ipomoea carnea*, have been scientifically validated for their anti-inflammatory, analgesic, and antioxidant activities (Galani et al. 2010; Zankar 2024). Despite its traditional use, *A. acuta* remains understudied, with limited empirical evidence supporting its pharmacological efficacy. Phytochemical analyses suggest that this species is rich in bioactive constituents such as flavonoids (kaempferol, rutin, apigenin), phenolic acids (gallic acid, caffeic acid), alkaloids (berberine), terpenoids (limonene), and saponins – compounds known to modulate inflammatory pathways, inhibit prostaglandin synthesis, and attenuate nociceptive responses (Li et al. 2021; Huang et al. 2022; Wijesekara et al. 2024). These biochemical properties indicate that *A. acuta* may possess significant therapeutic potential for managing inflammation-related disorders.

Nevertheless, the lack of detailed pharmacological studies, particularly those focusing on the ethanol extract of *A. acuta* leaves, limits the current scientific understanding of its medicinal potential. With growing interest in plant-based therapeutics due to their perceived safety and efficacy, comprehensive investigations into *A. acuta* are warranted. Such studies could provide foundational data for developing novel, plant-derived therapeutic agents.

The present study aims to evaluate the analgesic, antipyretic, and anti-inflammatory effects of ethanol extract from *A. acuta* leaves using well-established murine models. By elucidating the extract’s biological activities, this research seeks to validate the plant’s traditional uses and explore its potential as a natural alternative for treating pain, fever, and inflammation.

## Materials and methods

### Acquisition of plant material and extraction procedures

*A. acuta* leaves were collected in April 2024 from the Son Tra area, Quang Ngai Province, Vietnam. The harvested plant material was initially inspected to remove damaged or unsuitable samples, followed by thorough washing with distilled water to eliminate surface contaminants. The cleaned leaves were then shade-dried for two consecutive days to avoid direct sunlight exposure, thereby preserving thermolabile and photosensitive phytochemicals. After drying, the leaves were ground into a fine powder using an MRC Laboratory Grinder (MRC Ltd., Israel) and stored in dry, well-ventilated conditions until further processing.

For extraction, 200 g of the powdered leaf material was macerated in 2,000 ml of absolute ethanol. The mixture was subjected to ultrasound-assisted extraction at a frequency range of 40–60 kHz for 30–60 min, with intermittent stirring to enhance the efficiency of bioactive compound release. Following extraction, the mixture was filtered through muslin cloth to remove solid residues, yielding a clear filtrate. The crude extract was then concentrated under reduced pressure at 50°C using a rotary evaporator (Heidolph Instruments GmbH & Co. KG, Germany) to remove residual ethanol. The final ethanol extract of *A. acuta* leaves (AAEE) was obtained with a yield of 28% and stored in amber-colored, airtight containers at 4°C to ensure chemical stability and prevent degradation before further experimental use.

### Phytochemical profiling of Argyreia acuta extract

The preliminary phytochemical composition of the ethanol extract of AAEE was assessed through standard qualitative assays based on colorimetric and precipitation reactions with specific reagents. The presence of major phytochemical classes – including tannins, flavonoids, terpenoids, polyphenols, saponins, steroids, alkaloids, and cardiac glycosides – was determined via characteristic color changes or precipitate formation upon interaction with corresponding chemical agents, as described by Tran et al. (2023a).

Quantitative analysis of selected phytochemicals (flavonoids, alkaloids, and tannins) in AAEE was performed using spectrophotometric methods. Flavonoid content was measured via the aluminum chloride colorimetric assay, in which the extract reacts with AlCl_3_ to form a yellow-to-orange complex, and absorbance was recorded at 415 nm. Alkaloid content was determined by reaction with Mayer’s reagent, producing a white precipitate; the resulting solution was measured spectrophotometrically at 280 nm. Tannin content was quantified by reacting the extract with FeCl_3_, forming a blue-black complex, with absorbance measured at 765 nm (Nhung and Quoc, 2024a). These methods ensured reliable quantification of key bioactive constituents in the extract.

### Animal experiments

Healthy male Swiss albino mice (30 ± 2 g) were procured from the Pasteur Institute, Ho Chi Minh City, Vietnam. Upon arrival, the animals underwent a 7-day acclimatization period under standardized laboratory conditions. During this time, mice were housed in polypropylene cages lined with rice husks, which were regularly treated with a biological deodorizing agent to control odor. Environmental parameters were maintained at a temperature of 24 ± 2°C, relative humidity of 55 ± 5%, and a 12-h light/dark cycle. Mice were provided with a commercial rodent pellet diet and filtered drinking water ad libitum.

All animal handling and experimental protocols strictly adhered to the ethical guidelines set forth by the International Council for Laboratory Animal Science (ICLAS 2012) and complied with the ARRIVE 2.0 guidelines for reporting animal research (Percie du Sert et al. 2020).

### Experimental design

Sample sizes were estimated based on prior literature and refined using power analysis to ensure statistical robustness while adhering to the principles of the 3Rs (Replacement, Reduction, and Refinement). Animals were randomly assigned to experimental groups using a computerized randomization tool to minimize allocation bias. Blinding was implemented throughout data collection and analysis to reduce the risk of subjective bias (Rajput et al. 2023).

### Analgesic activity

#### Hot-plate assay

The hot plate assay was utilized to assess central analgesic effects by exposing the animal to a consistently heated surface and recording their latency to respond. Behavioral indicators such as paw licking, lifting, or jumping were observed when mice were placed on a hot plate (VELP^®^, Italy) maintained at 50 ± 2°C.

A total of 25 mice were fasted for 12 h with free access to water and randomly divided into five groups, each comprising five individuals. The control group received normal saline (10 ml/kg), while the standard group (TRM) was administered tramadol at 5 mg/kg. The remaining groups were treated with AAEE at doses of 100, 150, and 200 mg/kg (AAEE100, AAEE150, AAEE200). Reaction times were recorded at 0, 15, 30, 45, and 60 min before and after treatment.

To prevent potential tissue damage, the maximum response time was capped at 45 s. Throughout the assay, animals were closely monitored for any signs of discomfort or distress and were immediately removed from the apparatus upon responding. All procedures were conducted under strict ethical oversight (Nhung and Quoc 2024b).

The maximum analgesic effect in the hot plate test (AHP) was calculated using the following formula:


$$\text{AHP}\;[\%]=\frac{\text{Test reaction time}\;–\;\text{Control reaction time}}{45\;–\;\text{Control reaction time}}\times 100$$

#### Tail-flick assay

The tail-flick assay is a widely recognized method for evaluating analgesic effectiveness, wherein pain is induced through thermal stimulation by immersing the distal segment of the animal’s tail in heated water. In this study, 25 mice were used, each subjected to a 12-h fasting period with unrestricted access to water. The animals were randomly assigned to five groups, with each group comprising five mice.

The control group received normal saline (10 ml/kg), while the standard treatment group (TRM) was administered tramadol at a dose of 5 mg/kg. The remaining three groups were treated orally with AAEE at doses of 100, 150, and 200 mg/kg (AAEE100, AAEE150, AAEE200). During the experiment, each mouse was gently restrained in a device with its tail extended outward. The distal 1–3 cm segment of the tail was then submerged in hot water maintained at 50–55°C.

Reaction time, defined as the latency (in seconds) from tail immersion to withdrawal, was recorded before and after treatment at intervals of 0, 30, and 60 min. To minimize the risk of tissue damage, a maximum response latency of 15 s was enforced. Mice were carefully observed throughout the procedure, and any animal that responded was immediately removed from the testing setup. All experimental protocols were conducted in full compliance with strict ethical guidelines (Nhung and Quoc 2024c).

The percentage of pain inhibition in the tail-flick test (PPT) was calculated using the following formula:


$$\text{PPT}\;[\%]=\frac{\text{Test latency}\;–\;\text{Control latency}}{15\;–\;\text{Control latency}}\times 100$$

### Antipyretic activity

The antipyretic efficacy of AAEE was evaluated using a yeast-induced fever model with a 20% yeast suspension, following a standardized protocol with minor modifications (Nhung and Quoc 2024d). In this study, thirty mice were randomly assigned to six groups, each consisting of five animals.

The control group received sterile saline (10 ml/kg) without fever induction. The yeast group was administered a yeast solution (10 ml/kg) to induce hyperthermia but did not receive any subsequent treatment. The yeast plus Paracetamol group (Yeast+PCM) included mice treated with the yeast solution (10 ml/kg) followed by oral administration of Paracetamol at a dose of 150 mg/kg. The remaining three groups – Yeast+AAEE100, Yeast+ AAEE150, and Yeast+AAEE200 – received oral doses of AAEE at 100, 150, and 200 mg/kg, respectively, following yeast-induced fever (10 ml/kg).

Throughout the experiment, mice were observed for signs of discomfort, including lethargy, piloerection, and abnormal posturing. Any animal exhibiting severe symptoms was immediately euthanized by the humane endpoint criteria outlined in the ethical protocol.

### Evaluation of antipyretic activity via rectal temperature measurement

Before the experiment, the test animals were subjected to overnight fasting with unrestricted access to water. Fever was induced by subcutaneous administration of a 20% yeast solution at a dosage of 10 ml/kg body weight. Baseline rectal temperatures were recorded using a digital thermometer (Microlife, Microlife Corporation, Switzerland).

Eighteen hours after yeast injection, animals exhibiting an increase in rectal temperature ranging from 0.3 to 0.5°C were selected for antipyretic evaluation. The rectal temperatures of these animals were subsequently measured at 1, 2, and 3 h following treatment (Nhung and Quoc 2024d).

The percentage of fever reduction (PFR) was calculated using the following formula:


$$\text{PFR}\;[\%]=\frac{{\text{T}}_{\text{inital}}–{\text{T}}_{\text{post}–\text{treatment}}}{{\text{T}}_{\text{inital}}–{\text{T}}_{\text{baseline}}}\times 100$$

### Assessment of cyclooxygenase-2 and prostaglandin E_2_ levels

Blood samples were collected via the submandibular vein under light isoflurane anesthesia to minimize distress, by refinement principles. The collected blood was centrifuged at 12,000 rpm for 5 min to separate the serum. The concentrations of cyclooxygenase-2 (COX-2) and prostaglandin E_2_ (PGE_2_) were quantified using ELISA kits provided by Absolute Biotech Co., Ltd.

Serum samples and standard solutions were added to ELISA plates precoated with specific antibodies against COX-2 and PGE_2_. Horseradish peroxidase (HRP) conjugate was then added, and the plates were incubated at 37°C for 1 h. After incubation, the wells were washed three times with wash buffer to remove unbound substances.

Each assay was performed independently and in triplicate to ensure reproducibility. Reagents A and B were subsequently added and incubated at 37°C for an additional 15–30 min. The enzymatic reaction was terminated by the addition of a stop solution. Absorbance was measured at 450 nm using an enzyme-linked immunosorbent assay (ELISA) reader. The concentrations of COX-2 and PGE_2_ in serum samples were determined by comparing the optical density values to those from standard calibration curves (Nhung and Quoc 2023a).

### Anti-inflammatory activity

The carrageenan-induced paw edema model was employed to assess the anti-inflammatory effects of AAEE. Thirty Swiss mice were randomly divided into six groups, each comprising five animals (*n* = 5). The control group received an intraperitoneal injection of sterile saline (10 ml/kg) without inflammation induction. The carrageenan group (CGN) was administered a subcutaneous injection of 50 µl of 1% carrageenan solution to induce inflammation, without any subsequent treatment. The carrageenan plus indomethacin group (CGN+IND) received the carrageenan injection (50 µl of 1% solution) followed by an oral dose of indomethacin at 10 mg/kg. The three experimental groups (CGN+AAEE100, CGN+AAEE150, and CGN+AAEE200) received the same carrageenan injection, followed by oral administration of AAEE at doses of 100, 150, and 200 mg/kg, respectively. Throughout the study, mice were carefully monitored for indicators of discomfort, including reduced activity, fur bristling, and abnormal body posture. Animals exhibiting significant distress were promptly euthanized by the humane endpoint guidelines specified in the approved ethical protocol.

#### Evaluation of anti-inflammatory activity through paw circumference

The circumference of the right hind paw was measured using digital calipers (Fowler, Fowler High Precision, Inc., USA) immediately before the induction of inflammation. This initial measurement served as a baseline for evaluating edema severity and the antiinflammatory efficacy of the test substances.

Paw edema was quantified by recording paw circumference at specific time points: 0, 1, 2, 3, 4, and 5 h after carrageenan administration. Measurements were taken with high-precision digital calipers to accurately detect changes in paw size due to inflammation. The data obtained provided a comprehensive view of the progression of the inflammatory response and the impact of each treatment (Nhung and Quoc 2024d).

The percentage inhibition of paw edema (PPE), used as an indicator of anti-inflammatory efficacy, was calculated using the following standard formula:


$$\text{PPE}\;[\%]=\frac{{\text{Circumference}}_{\text{control}}\;–\;{\text{Circumference}}_{\text{treated}}}{{\text{Circumference}}_{\text{treated}}\;–\;{\text{Circumference}}_{\text{baseline}}}\times 100$$ [Eqn 1]

#### Assessment of cytokine concentrations

The concentrations of cytokines IL-6, TNF-α, and IL-1β were quantified using enzyme-linked immunosorbent assay (ELISA) combined with immunoassay techniques. In this procedure, specific antibodies targeting IL-6, TNF-α, and IL-1β were immobilized onto the wells of a 96-well plate. The plates were incubated overnight with the samples to allow the antigens to bind to their respective antibodies.

On the following day, biotinylated secondary antibodies were added to each well after incubation with either tissue samples or antigen standards. Subsequently, streptavidin-conjugated enzymes were introduced to facilitate a colorimetric reaction that changed the substrate color from purple to yellow. Absorbance was measured at a wavelength of 450 nm using an ELISA reader to determine the levels of IL-6, TNF-α, and IL-1β. Cytokine concentrations were calculated and expressed as ng/mg (Nhung and Quoc 2023b).

### Histopathological analysis

At the end of the experiment, mice were euthanized by gradual exposure to CO_2_ in a dedicated chamber until complete loss of consciousness, followed by cervical dislocation to ensure death. The inflamed paw tissues were collected, fixed in 10% formalin, and processed through standard histological procedures, including dehydration, clearing, paraffin embedding, and sectioning (4–5 µm).

The tissue sections were stained with Hematoxylin and Eosin (H&E) and examined microscopically to evaluate neutrophil infiltration, edema, vascular dilation, and tissue damage.

### Statistical analysis

Analysis of variance (ANOVA) was employed to assess variations in experimental parameters. Results are expressed as mean ± SD, with statistical significance set at *p* < 0.05. All statistical analyses were performed using Statgraphics Centurion XX.

## Results and discussion

### Phytochemical analysis and anti-inflammatory potential of Argyreia acuta ethanol extract

Phytochemical analysis of the AAEE confirmed the presence of tannins, flavonoids, terpenoids, polyphenols, saponins, steroids, and alkaloids, while cardiac glycosides were absent. The quantitative assessment revealed polyphenols as the predominant constituents (70.46 ± 1.42 mg GAE/g), followed by flavonoids (41.75 ± 1.16 mg QE/g) and tannins (7.78 ± 0.24 mg TE/g) (Table 1).

**Table 1:** Phytochemical screening and quantification of ethanol extract from *Argyreia acuta* leaves

Phytoconstituents	Test	Observation	Present in AAEE	Quantification of phytochemicals
Tannins	2 ml AAEE + 2 ml H_2_O + 2–3 drops FeCl_3_ (5%)	Green precipitate	+	7.78 ± 0.24 mg TE/g
Flavonoids	1 ml AAEE + 1 ml Pb(OAc)_4_ (10%)	Yellow coloration	+	41.75 ± 1.16 mg QE/g
Terpenoids	2 ml AAEE + 2 ml (CH_3_CO)_2_O + 2–3 drops conc. H2SO4	Deep red coloration	+	NT
Polyphenol	2 ml AAEE + 2 ml FeCl_3_	Bluish-green appearance	+	70.46 ± 1.42 mg GAE/g
Saponins	5 ml AAEE + 5 ml H_2_O + heat	Froth appears	+	NT
Steroids	2 ml AAEE + 2 ml CHCl_3_ + 2 ml H_2_SO_4_ (conc.)	The reddish-brown ring at the junction	+	NT
Cardiac glycosides	2 ml AAEE + 2 ml CHCl_3_ + 2 ml CH_3_COOH	Violet to blue to green coloration	–	–
Alkaloids	2 ml AAEE + a few drops of Hager’s reagent	Yellow precipitate	+	NT

Phytochemicals in AAEE are (+) present, (–) absent, and (NT) not tested

The diversity of phytochemicals present in AAEE suggests significant anti-inflammatory potential. The high concentrations of polyphenols and flavonoids are particularly noteworthy, as these compounds are recognized for their antioxidant and anti-inflammatory activities. They inhibit key pro-inflammatory mediators such as nuclear factor kappa B (NF-κB), cyclooxygenase (COX), and lipoxygenase (LOX), thereby reducing the production of cytokines like TNF-α, IL-1β, and IL-6. This suppression mitigates inflammation by decreasing oxidative stress and preventing immune cell activation (Intharuksa et al. 2024).

Tannins contribute by stabilizing cell membranes, reducing capillary permeability, and inhibiting histamine release, thus limiting inflammatory cell infiltration (Molnar et al. 2024). Terpenoids inhibit the synthesis of nitric oxide (NO) and prostaglandins (PGs) by downregulating inducible nitric oxide synthase (iNOS) and COX-2. Saponins enhance macrophage function and reduce inflammatory cell migration (Nhung and Quoc 2025). Steroids found in AAEE suppress inflammatory gene expression and help minimize tissue damage (Ferreira et al. 2024). Additionally, alkaloids exert antiinflammatory effects by modulating mitogen-activated protein kinases (MAPKs) and NF-κB signaling pathways (Tran and Tran 2021).

Previous studies have identified various phytochemicals in species of the *Argyreia* genus and assessed their anti-inflammatory potential. For instance, research on the botanical characteristics of *A. acuta* has provided a foundation for further investigation into its chemical composition and biological activities (Li et al. 2021). Additionally, studies on other medicinal *Argyreia* species have offered valuable insights into their phytochemical profiles (Zankar 2024; Galani et al. 2010). These findings demonstrate that *Argyreia* species contain a diverse array of bioactive compounds, supporting their potential application in anti-inflammatory therapies.

### Analgesic potential of AAEE

#### Hot-plate assay

**Figure 1:**
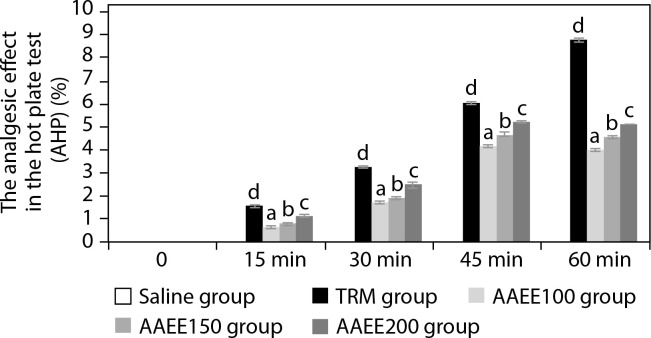
Time-dependent analgesic effects of ethanol extract from *Argyreia acuta* leaves in hot plate test. Results are expressed as mean ± SD, with letters (a, b, c, and d) indicating statistically significant group differences (*p* < 0.05)

Table 2 and Figure 1 illustrate the analgesic efficacy of AAEE in the hot-plate assay. The extract exhibited dose-dependent analgesic activity, with significantly enhanced paw-licking latency and AHP% at higher doses compared to the saline-treated control group (*p* < 0.05). The saline group showed no significant changes in paw-licking latency or pain inhibition percentage (AHP%) at any time point, confirming the absence of an analgesic effect.

**Table 2:** Effects of ethanol extract from *Argyreia acuta* leaves on reaction time in hot plate test

Time	Saline group	TRM group	AAEE100 group	AAEE150 group	AAEE200 group
0 min	4.52 ± 0.06^a^	4.62 ± 0.05^b^	4.55 ± 0.07^a^	4.53 ± 0.04^a^	4.54 ± 0.02^a^
15 min	4.45 ± 0.04^a^	5.08 ± 0.02^e^	4.71 ± 0.06^b^	4.77 ± 0.02^c^	4.91 ± 0.06^d^
30 min	4.21 ± 0.03^a^	5.54 ± 0.02^e^	4.91 ± 0.03^b^	4.99 ± 0.02^c^	5.22 ± 0.06^d^
45 min	3.99 ± 0.03^a^	6.47 ± 0.02^e^	5.68 ± 0.05^b^	5.91 ± 0.06^c^	6.13 ± 0.04^d^
60 min	3.81 ± 0.03^a^	7.42 ± 0.04^e^	5.45 ± 0.04^b^	5.68 ± 0.04^c^	5.91 ± 0.03^d^

Values are expressed as mean ± SD and letters (a, b, c, d, and e) represent the difference between groups (*p* < 0.05)

In contrast, the standard treatment group (TRM) displayed a substantial and sustained increase in paw-licking latency, reaching 7.42 ± 0.04 s and an AHP% of 8.77% at 60 min (*p* < 0.05), indicating robust central analgesic activity. Among AAEE-treated groups, both latency and AHP% increased in a dose-dependent manner.

The 100 mg/kg dose produced moderate effects, with latency peaking at 45 minutes (5.68 ± 0.05 s) and AHP% reaching 4.13%, followed by a slight decline at 60 min. The 150 and 200 mg/kg doses induced more pronounced and sustained analgesic effects, with latencies of 5.91 ± 0.06 s and 6.13 ± 0.04 s and corresponding AHP% values of 4.68% and 5.21% at 45 min, respectively. These higher doses continued to show significant analgesic activity at 60 min compared to the saline group (*p* < 0.05).

#### Tail-flick assay

**Figure 2:**
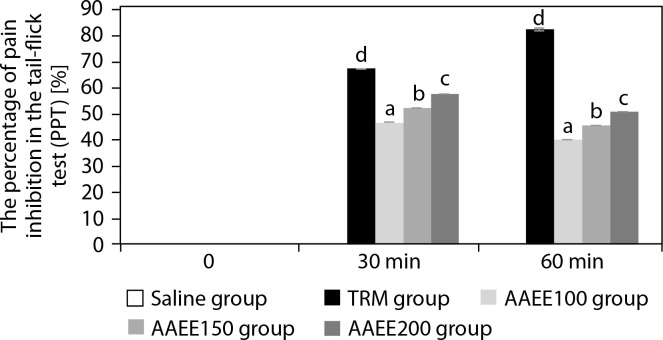
Percentage of pain inhibition in tail-flick assay over time. Results are expressed as mean ± SD, with letters (a, b, c, and d) indicating statistically significant group differences (*p* < 0.05)

Table 3 and Figure 2 depict the analgesic effects of AAEE in the tail-flick test, revealing dose-dependent efficacy with significant improvements over the saline control (*p* < 0.05). The saline group showed no significant changes in tail-flick latency or pain percentage threshold (PPT%), confirming the absence of analgesic activity. In contrast, the TRM group demonstrated strong and sustained analgesic effects, reaching a tailflick latency of 13.52 ± 0.04 s and a PPT% of 82.43% at 60 min (*p* < 0.05).

**Table 3:** Time-dependent changes in reaction time during tail-flick assay across different treatment groups

Time	Saline group	TRM group	AAEE100 group	AAEE150 group	AAEE200 group
0 min	7.50 ± 0.04^a^	7.51 ± 0.05^a^	7.40 ± 0.03^a^	7.44 ± 0.05^a^	7.50 ± 0.03^a^
30 min	7.00 ± 0.04^a^	12.38 ± 0.03^e^	10.73 ±0.03^b^	11.18 ± 0.03^c^	11.63 ± 0.03^d^
60 min	6.60 ± 0.02^a^	13.52 ± 0.04^e^	9.99 ± 0.02^b^	10.43 ± 0.02^c^	10.88 ± 0.02^d^

Values are expressed as mean ± SD and letters (a, b, c, d, and e) represent the difference between groups (*p* < 0.05)

AAEE-treated groups exhibited dose-dependent increases in tail-flick latency and PPT%. The 100 mg/kg dose peaked at 30 min (10.73 ± 0.03 s, PPT%: 46.60%) but declined by 60 min. The 150 mg dose reached 11.18 ± 0.03 s and 52.25% PPT at 30 min, followed by a slight reduction. The 200 mg/kg dose displayed the highest efficacy, with a latency of 11.63 ± 0.03 s and a PPT% of 57.83% at 30 min, maintaining elevated values at 60 min compared to lower doses (*p* < 0.05). These findings support the analgesic potential of AAEE, particularly at 150 and 200 mg doses.

The analgesic effects of AAEE are likely mediated through the inhibition of inflammatory enzymes COX-1 and COX-2, thereby reducing prostaglandin synthesis – an essential mediator of pain (Iolascon et al. 2021). In addition, AAEE contains flavonoids and polyphenols, which act as antioxidants, mitigating oxidative stress in the nervous system by scavenging free radicals and reducing pain perception (Nhung and Quoc 2024a). Interaction with central opioid receptors may further inhibit pain signal transmission, similar to the mechanism of centrally acting analgesics (Nhung and Quoc 2024b).

The combination of anti-inflammatory and central analgesic mechanisms enhances AAEE’s pain-relieving potential. Notably, the sustained effects observed at 150 mg suggest that the prolonged release or metabolism of active constituents contributes to its efficacy. These results are consistent with previous studies on *A. speciosa* and *A. argentea*, which also demonstrated significant analgesic activity in hot-plate tests via inhibition of inflammatory mediators, antioxidant activity, and opioid receptor interactions (Lalan et al. 2015; Dina et al. 2010). This consistency underscores the therapeutic promise of the *Argyreia* genus as a source of natural analgesics and validates AAEE’s potential as an effective analgesic agent.

### Antipyretic properties of AAEE

#### Evaluation of antipyretic activity via rectal temperature measurement

**Figure 3:**
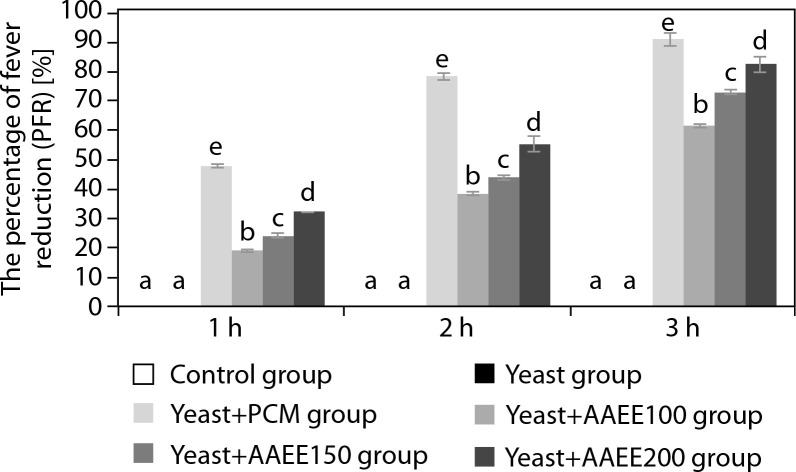
Percentage of fever reduction over time in the antipyretic assay. Results are expressed as mean ± SD, with letters (a, b, c, d, and e) indicating statistically significant group differences (*p* < 0.05)

Table 4 and Figure 3 demonstrate that AAEE exhibits significant antipyretic effects in a yeast-induced fever model in mice, with efficacy increasing in a dose-dependent manner (*p* < 0.05). The group administered yeast to induce pyrexia showed a marked rise in rectal temperature, peaking at 39.45°C after 3 h (*p* < 0.05). The reference drug reduced rectal temperature to 36.91°C, corresponding to a fever inhibition rate of 91.06% after 3 h.

**Table 4:** Changes in rectal temperature over time to assess antipyretic effects of AAEE in yeast-induced fever model

Experimental group	Initial [°C]	Fever [°C]	1 h [°C]	2 h [°C]	3 h [°C]
Control group	36.78 ± 0.03^a^	36.84 ± 0.05^a^	36.82 ± 0.04^a^	36.79 ± 0.03^a^	36.81 ± 0.02^a^
Yeast group	36.51 ± 0.05^a^	39.11 ± 0.04^d^	39.31 ± 0.04^f^	39.41 ± 0.04^f^	39.45 ± 0.04^f^
Yeast+PCM group	36.71 ± 0.01^c^	38.98 ± 0.05^a^	37.89 ± 0.01^a^	37.21 ± 0.04^a^	36.91 ± 0.04^a^
Yeast+AAEE100 group	36.61 ± 0.04^b^	39.21±0.06^c^	38.71±0.05^d^	38.21±0.03^d^	37.51 ± 0.03^d^
Yeast+AAEE150 group	36.61 ± 0.04^b^	39.11 ± 0.05^d^	38.51 ± 0.04^e^	38.01 ± 0.06^e^	37.28 ± 0.05^e^
Yeast+AAEE200 group	36.49 ± 0.04^a^	39.01 ± 0.04^b^	38.19 ± 0.02^c^	37.61 ± 0.05^c^	36.93 ± 0.04^c^

Values are expressed as mean ± SD and letters (a, b, c, d, e, and f) represent the difference between groups (*p* < 0.05)

AAEE-treated groups exhibited significant reductions in rectal temperature compared to the untreated yeast-induced group. At 100 mg/kg, AAEE lowered the temperature from 39.21 to 37.51°C after 3 h, with inhibition rates of 19.14, 38.28, and 61.48% at 1, 2, and 3 h, respectively. The 150 mg/kg dose reduced the temperature to 37.28°C, with corresponding inhibition rates of 24.04, 43.86, and 72.85%. The 200 mg/kg dose produced the most pronounced antipyretic effect, reducing the temperature to 36.93°C, with inhibition rates of 32.08, 55.30, and 82.61% at the respective time points—closely approximating PCM’s efficacy.

### Assessment of cyclooxygenase-2 and prostaglandin E_2_ levels

**Figure 4:**
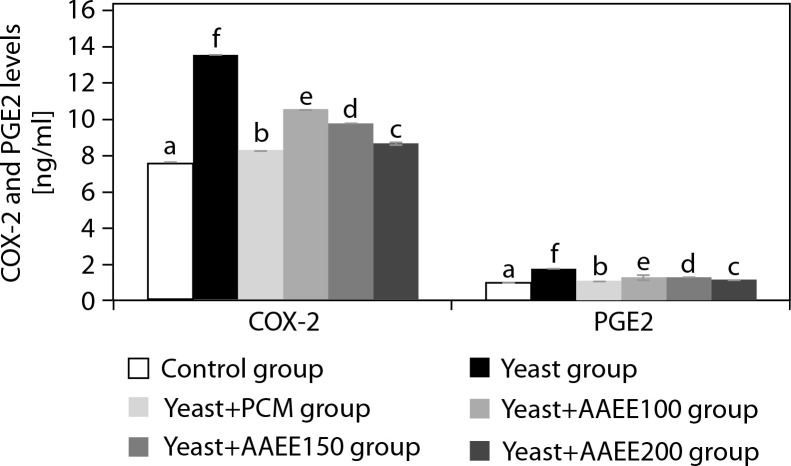
Effects of ethanol extract from *Argyreia acuta* leaves and PCM on COX-2 and PGE_2_ levels in antipyretic assay. Results are expressed as mean ± SD, with letters (a, b, c, d, e, and f) indicating statistically significant group differences (*p* < 0.05)

Figure 4 illustrates that AAEE significantly reduced COX-2 and PGE_2_ levels in yeast-induced fever, supporting its anti-inflammatory and antipyretic properties. The yeast group showed elevated COX-2 and PGE_2_ levels (13.55 and 1.75 ng/ml) compared to the control group (7.53 and 0.97 ng/ml). PCM treatment lowered these levels to 8.29 and 1.07 ng/ml, respectively. AAEE reduced COX-2 and PGE_2_ in a dose-dependent manner: 100 mg/kg decreased levels to 10.54 and 1.27 ng/ml; 150 mg/kg to 9.79 and 1.27 ng/ml; and 200 mg/kg to 8.67 and 1.12 ng/ml, approaching PCM’s effects.

AAEE’s antipyretic activity is attributed to the modulation of inflammatory pathways, as evidenced by lowered rectal temperatures and reduced COX-2 and PGE_2_ concentrations. By inhibiting the COX-2 pathway, AAEE decreases PGE_2_ synthesis, thereby stabilizing hypothalamic temperature regulation (Kulesza et al. 2023). Phytochemicals in AAEE – such as flavonoids, alkaloids, and phenolic compounds – likely act as COX-2 inhibitors or antioxidants, reducing oxidative stress and systemic inflammation (Nguyen et al. 2021). This dual mechanism aligns AAEE’s efficacy with that of PCM, positioning it as a promising natural alternative for fever management.

These findings are consistent with previous studies on *A. speciosa* and other medicinal plants such as *Caryota urens* and *Plukenetia volubilis*, which demonstrated antipyretic effects through similar COX–PGE_2_ pathway modulation (Nhung and Quoc 2024a, 2024c; Lalan et al. 2015). The consistency across species reinforces *A. acuta*’s therapeutic potential as a natural antipyretic agent and supports its traditional use in ethnomedicine for fever treatment. Further phytochemical and mechanistic studies are warranted to fully elucidate AAEE’s medicinal properties.

### Anti-inflammatory properties of AAEE

#### Anti-inflammatory effects in carrageenan-induced paw edema model

**Figure 5:**
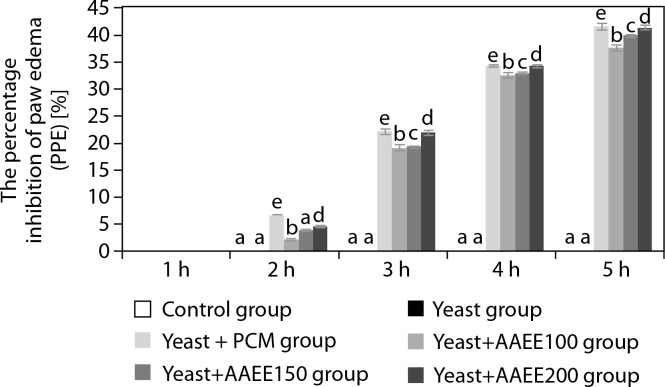
Percentage inhibition of paw edema in carrageenaninduced inflammation in mice following treatment with ethanol extract of *Argyreia acuta*. Results are expressed as mean ± SD, with letters (a, b, c, d, and e) indicating statistically significant group differences (*p* < 0.05)

Table 5 and Figure 5 illustrate the effects of AAEE on carrageenan-induced paw inflammation in mice, as assessed by changes in paw circumference and percentage paw edema inhibition (PPE%). The control group maintained a stable paw circumference (22.63– 22.71 mm) and exhibited no inflammation inhibition (PPE% = 0). In contrast, the carrageenan group (CGN) displayed a significant increase in paw circumference, peaking at 39.73 mm at 4 h, thereby confirming the successful induction of inflammation. Treatment with indomethacin (CGN+IND) resulted in a marked reduction in paw swelling, from 29.44 at 1 h to 23.19 mm at 4 h, and a corresponding increase in PPE% from 6.68 to 41.62%, demonstrating strong anti-inflammatory activity.

**Table 5:** Effects of ethanol extract from *Argyreia acuta* on paw edema thickness in carrageenan-induced inflammation in mice

Experimental group	“0” [mm]	1 h [mm]	2 h [mm]	3 h [mm]	4 h [mm]
Control group	22.63 ± 0.19^a^	22.69 ± 0.19^a^	22.68 ± 0.13^a^	22.71 ± 0.24^a^	22.66 ± 0.19^a^
CGN group	22.68 ± 0.21^a^	31.55 ± 0.28^f^	34.73 ± 0.29^e^	37.68 ± 0.31^e^	39.73 ± 0.32^d^
CGN+IND group	22.65 ± 0.20^a^	29.44 ± 0.26^d^	27.04 ± 0.23^d^	24.75 ± 0.22^d^	23.19 ± 0.26^a^
CGN+AAEE100 group	22.65 ± 0.21^a^	30.87 ± 0.27^e^	28.08 ± 0.24^c^	25.40 ± 0.22^d^	24.77 ± 0.14^c^
CGN+AAEE150 group	22.63 ± 0.20^a^	30.34 ± 0.26^b^	28.08 ± 0.04^c^	25.32 ± 0.07^c^	23.92 ± 0.11^b^
CGN+AAEE200 group	22.64 ± 0.21^a^	30.11 ± 0.27^c^	27.19 ± 0.15^b^	24.83 ± 0.12^b^	21.53 ± 0.21^a^

Values are expressed as mean ± SD and letters (a, b, c, d, e, and f) represent the difference between groups (*p* < 0.05)

AAEE exhibited a clear dose-dependent inhibition of inflammation. At 100 mg/kg, paw circumference decreased from 30.87 to 24.77 mm, with a PPE% of 37.65%. A dose of 150 mg/kg further improved the antiinflammatory response, resulting in a PPE% of 39.93%. The highest dose of 200 mg/kg reduced paw circumference to 23.92 mm and achieved a PPE% of 41.39%, a value closely matching that of the indomethacin-treated group.

### Cytokine level modulation by AAEE

Table 6 presents the concentrations of pro-inflammatory cytokines TNF-α, IL-1β, and IL-6 following carrageenan-induced inflammation. The control group exhibited the lowest levels of these cytokines, with TNF-α at 142.16 pg/ml, IL-1β at 276.24 pg/ml, and IL-6 at 27.11 pg/ml, consistent with the absence of inflammation. In contrast, the carrageenan group showed a marked elevation in cytokine levels, with TNF-α increasing to 255.89 pg/ml, IL-1β to 497.23 pg/ml, and IL-6 to 48.80 pg/ml, confirming a strong inflammatory response.

**Table 6:** Effects of ethanol extract from *Argyreia acuta* on pro-inflammatory cytokine levels in carrageenan-induced inflam-mation in mice

Experimental group	TNF-α [pg/ml]	IL-1β [pg/ml]	IL-6 [pg/ml]
Control group	142.16 ± 1.26^a^	276.24 ± 2.06^a^	27.11 ± 0.14^a^
CGN group	255.89 ± 1.73^f^	497.23 ± 4.25^f^	48.80 ± 0.24^f^
CGN+IND group	156.37 ± 1.40^b^	303.86 ± 2.72^b^	29.82 ± 0.16^b^
CGN+AAEE100 group	213.24 ± 1.68^e^	414.36 ± 3.81^e^	40.66 ± 0.23^e^
CGN+AAEE150 group	199.02 ± 1.50^d^	386.74 ± 3.50^d^	37.95 ± 0.21^d^
CGN+AAEE200 group	170.59 ± 1.44^c^	331.49 ± 3.16^c^	32.53 ± 0.18^c^

Values are expressed as mean ± SD and letters (a, b, c, d, e, and f) represent the difference between groups (*p* < 0.05)

Treatment with indomethacin significantly suppressed cytokine levels, with TNF-α reduced to 156.37 pg/ml, IL-1β to 303.86 pg/ml, and IL-6 to 29.82 pg/ml. AAEE demonstrated a similar dose-dependent cytokine suppression. At 100 mg/kg, TNF-α, IL-1β, and IL-6 levels decreased to 213.24, 414.36, and 40.66 pg/ml, respectively. The 150 mg/kg dose further reduced these levels to 199.02, 386.74, and 37.95 pg/ml. The highest dose of 200 mg/kg showed the strongest suppressive effect, with TNF-α at 170.59 pg/ml, IL-1β at 331.49 pg/ml, and IL-6 at 32.53 pg/ml, approaching the values observed in the indomethacin-treated group.

### Morphological and histopathological analysis

**Figure 6:**
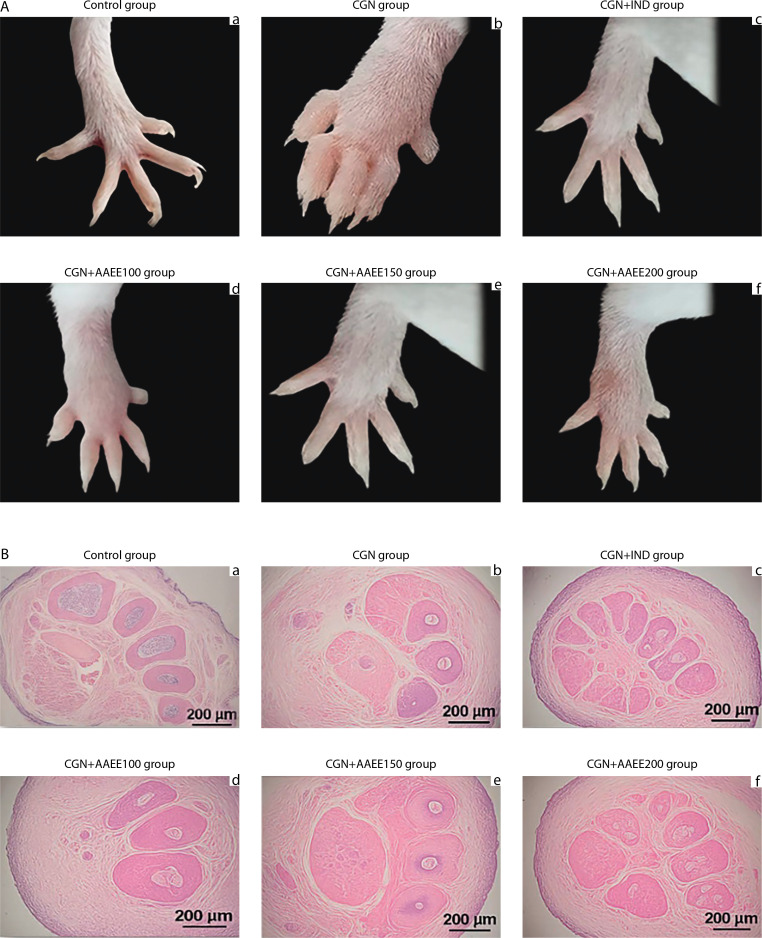
Effects of ethanol extract from *Argyreia acuta* on carrageenan-induced paw edema in mice. **A**) Representative macroscopic images of mouse paws from different experimental groups. **B**) Histological analysis of paw tissue sections stained with hematoxylin and eosin (H&E), magnification ×200

Figure 6A depicts rat paw morphology across the different treatment groups. The control group (Figure 6A-a) exhibited normal paw structure without signs of swelling, redness, or deformation. The toes were fully extended and well separated, indicating the absence of inflammation. In contrast, the carrageenan group (CGN; Figure 6A-b) showed pronounced swelling, redness, and edema, particularly in the joints and dorsal surface of the paw. Toes appeared contracted and less flexible, confirming the presence of severe inflammation. Indomethacin treatment (CGN+IND; Figure 6A-c) markedly reduced swelling and redness, nearly restoring normal paw morphology and toe separation, indicative of effective anti-inflammatory action.

AAEE at 100 mg/kg (CGN+AAEE100; Figure 6A-d) resulted in mild edema reduction, although some swelling, redness, and toe contraction remained, suggesting a moderate anti-inflammatory effect. The 150 mg/kg dose (CGN+AAEE150; Figure 6A-e) produced a more pronounced reduction in inflammation, with visibly decreased swelling and improved toe extension, indicating enhanced efficacy. The CGN+AAEE200 group (Figure 6A-f) demonstrated the strongest anti-inflammatory response among the AAEE-treated groups, with paw morphology nearly restored. Significant edema reduction and improved toe flexibility were observed, comparable to the effects of indomethacin.

Histological analysis further supported the morphological findings. The control group (Figure 6B-a) exhibited intact tissue architecture, with well-preserved hair follicles and connective tissue, and no signs of inflammation or edema. In contrast, the carrageenan group (CGN; Figure 6B-b) showed severe edema and inflammatory cell infiltration, along with disrupted tissue integrity. Additional features included reduced connective tissue density, swollen and irregular hair follicles, mild epithelial degeneration, dilated blood vessels, and separated muscle fibers with mild degeneration – hallmarks of significant inflammatory damage. Indomethacin treatment (CGN+IND; Figure 6B-c) resulted in notable recovery, with reduced edema and inflammation, although minor structural alterations persisted. AAEE at 100 mg/kg (CGN+AAEE100; Figure 6B-d) produced slight reductions in edema and inflammatory infiltration; however, the presence of loose connective tissue, dilated vessels, and swollen hair follicles suggested incomplete recovery. The 150 mg/kg, AAEE (CGN+AAEE150; Figure 6B-e) resulted in further improvement, with decreased edema, more organized connective tissue, and reduced inflammatory cell infiltration. The highest AAEE dose (CGN+AAEE200; Figure 6B-f) exhibited the most significant histological recovery, with minimal inflammation, restored hair follicle structure, and well-preserved tissue structure.

AAEE demonstrated significant anti-inflammatory effects, as evidenced by reduced paw edema, increased inflammation inhibition, suppression of pro-inflammatory cytokines, and improved tissue morphology. The data indicate a clear dose-dependent anti-inflammatory response, with higher doses (150–200 mg/kg) approaching the efficacy of indomethacin (IND), a widely used standard anti-inflammatory agent. CGN-induced inflammation occurs in two phases: the early phase (0–2 h), primarily mediated by histamine and serotonin, and the late phase (3–4 h), driven by prostaglandins and pro-inflammatory cytokines such as TNF-α, IL-1β, and IL-6 (Berrueta et al. 2023). AAEE significantly reduced paw circumference and increased PPE%, suggesting effective modulation of both phases of inflammation. The early-phase inhibition may result from mast cell stabilization or suppression of histamine release, while the late-phase activity likely involves interference with the arachidonic acid cascade, potentially through COX inhibition and suppression of prostaglandin synthesis (Tran et al. 2023b).

In addition, AAEE significantly reduced TNF-α, IL-1β, and IL-6 levels, indicating its ability to interfere with NF-κB signaling and downregulate the production of inflammatory mediators, similar to the known mechanism of indomethacin (Pal et al. 2023). Histopathological analysis confirmed these findings by demonstrating that AAEE mitigated tissue damage, reduced inflammatory cell infiltration, and preserved overall tissue structure (Santoso et al. 2024). Higher doses (150–200 mg/kg) effectively reduced edema, vascular congestion, and inflammatory infiltration, restoring connective tissue structure and hair follicle morphology. These results suggest that the antiinflammatory action of AAEE is mediated through multiple pathways, including the inhibition of prostaglandin and leukotriene synthesis via COX and LOX enzymes, antioxidant effects, and free radical scavenging activity that together help reduce oxidative stress and cytokine overproduction. The observed suppression of TNF-α and IL-1β further supports the notion that AAEE downregulates NF-κB signaling, thereby attenuating the inflammatory response (Zhang et al. 2022).

Numerous studies have validated the anti-inflammatory efficacy of herbal extracts by demonstrating reductions in edema, increases in inhibition percentages, suppression of pro-inflammatory cytokines, and improved histological profiles. For example, the ethanol extract of *Spondias mangifera* fruit significantly reduced joint swelling and cytokine levels (TNF-α and IL-6) in a CFA-induced arthritis model in rats, indicating strong anti-arthritic and anti-inflammatory properties (Khalid et al. 2021). Histopathological evaluation confirmed its efficacy through reductions in synovial inflammation and cartilage degradation, along with preservation of joint space integrity. Similarly, a standardized methanolic extract of *Muntingia calabura* leaves significantly reduced paw edema in a carrageenan-induced inflammation model in rats, further confirming its potent antiinflammatory activity (Jisha et al. 2019).

Furthermore, the ethanol extract of *Mahonia bealei* significantly inhibited pro-inflammatory cytokines TNF-α and IL-6 in vitro, underscoring its potential for inflammation management (Hu et al. 2016). Collectively, these findings support the conclusion that various herbal extracts exert anti-inflammatory effects by reducing edema, enhancing inflammation inhibition, downregulating pro-inflammatory cytokines, and improving histopathological features. These outcomes are consistent with the present study on AAEE, further reinforcing its potential as a natural anti-inflammatory agent.

## Conclusions

AAEE exhibits potent anti-inflammatory, analgesic, and antipyretic activities, which can be attributed to its rich phytochemical composition – including polyphenols, flavonoids, tannins, terpenoids, saponins, steroids, and alkaloids. AAEE effectively reduced carrageenaninduced paw edema and significantly suppressed proinflammatory cytokines (TNF-α, IL-1β, and IL-6), achieving results comparable to those of indomethacin. It also provided dose-dependent analgesia in both hot plate and tail-flick tests and effectively lowered yeast-induced fever. Histopathological analysis further confirmed AAEE’s ability to minimize tissue damage and inflammatory cell infiltration. Taken together, these findings highlight *A. acuta* as a promising natural therapeutic candidate for the management of inflammation, pain, and fever.
